# The Effect of Integrated Intervention Based on Protection Motivation Theory and Implementation Intention to Promote Physical Activity and Physiological Indicators of Patients with Type 2 Diabetes

**DOI:** 10.1155/2021/6637656

**Published:** 2021-06-22

**Authors:** Mohammad Ali Morowatisharifabad, Mohammad Asadpour, Mohammad Ali Zakeri, Mahdi Abdolkarimi

**Affiliations:** ^1^Department of Health Education and Promotion, School of Public Health, Shahid Sadoughi, University of Medical Sciences, Yazd, Iran; ^2^Department of Health Education and Health Promotion, School of Health, Rafsanjan University of Medical Sciences, Rafsanjan, Iran; ^3^Non-Communicable Diseases Research Center, Rafsanjan University of Medical Sciences, Rafsanjan, Iran; ^4^Determinants of Health Research Centre, Rafsanjan University of Medical Sciences, Rafsanjan, Iran

## Abstract

Despite benefits of physical activity, the level of physical activity is not desirable in patients with type 2 diabetes. The aim of this study is the using of integration of intervention based on the theory of protection motivation and implementation intention in order to improve the level of activity in patients with diabetes. This field trial study has been performed on 125 patients with type 2 diabetes. Samples have been randomly selected, and they are divided into two intervention and control groups. In the intervention group, training sessions were conducted based on the protection motivation theory and implementation intention. Physical activity levels, VO2 max, and hemoglobin A1C were measured before and three months after the intervention in the two groups. Data were analyzed by using SPSS 18, and independent *t*-test, paired *t*-test, and equivalent nonparametric tests were used for analyzing abnormal data. The results of this study showed that the level of physical activity was higher in the intervention group (*p* = 0.02). Also, the amount of hemoglobin A1c in the intervention group has been decreased significantly three months later (*p* < 0.001). In this study, VO2 max and blood lipids were not significantly different in the two groups. However, there was higher VO2 max compared to before the intervention in the intervention group. The present study showed that combining motivational interventions and implementing intention intervention can be effective in promoting the physical activity of patients with type 2 diabetes.

## 1. Introduction

*Diabetes* is a major public *health problem* that is approaching *at an alarming* rate. In 2019, nearly half a billion people were living with diabetes worldwide, which has increased 10.2 percent by 2030 [1]. In Iran, latest national survey estimated the national prevalence of diabetes at 11.4% of the adult population [2]. Physical activity, nutrition, drug therapy, and stress controlling are the four main methods of controlling diabetes and preventing its complications [3]. Promoting physical activity is one of the most important ways to manage and control diabetes and its complications, which is highly emphasized due to *easy access* and its cost-effectiveness [4]. Sports activity is not only effective in lowering blood sugar and increasing insulin secretion but also reduces the risk of cardiovascular disease and obesity, which are the most important causes of mortality and disability in this group [5]. Some studies show that lack of adequate physical activity is one of the most important risk factors for diabetes-related death, and physical activity reduces the risk and mortality in diabetic men by 3 to 3.5 percent [6]. People living with diabetes should engage in 150 minutes of moderate to vigorous intensity physical activity per week, spread over at least 3 days per week [7]. Although diabetics are often encouraged to exercise, they are usually less successful. Such that, some patients are unable to maintain their motivation to continue physical activity, and there are many personal and environmental barriers that cause instability in their physical activity [8]. As some studies show, sedentary lifestyle has been reported 60.1% in men with diabetes and 72.9% in women with diabetes [9]. Given this fact, it seems necessary to create appropriate interventions for physical activity in this group.

Today, training based on changing behavior theories has been accepted as the core of health care activities which can change patients' behavior [10]. The Rogers' Protection Motivation Theory is one of the proposed theories to promote health theory. This theory is based on conducting health-related behaviors which are associated with a person's motivation to protect them from potential danger [11]. According to this theory, people usually intend to accept the recommended health behaviors when their perceived threat is at the highest level (perceived vulnerability and perceived severity), and in the following, they should believe that the recommended behaviors are effective (response and self-efficacy) [12]. Some studies have shown that the theoretical structures of protective motivation can predict the behavior of physical activity in patients with diabetes [13]. Motivational theories consider intention or motivation as the most important determinant of behavior. Although intention is one of the important determinants of physical activity, some studies have shown that there is always a gap between intention and motivation to engage in physical activity [14]. According to this view, a process has been proposed by Gollwitzer which called *Implementation intention*. Based on this process, if people have plans and predictions for how to behave and remove obstacles, the possibility of turning motivation into behavior will increase [15]. According to this theory, two steps are necessary to turn the intention of behavior into action. The first stage of action planning is where the details of how the behavior will be performed, and the planning stage for adaptation is where strategies are anticipated to overcome possible barriers to behavior [16]. Therefore, considering the importance of promoting physical activity in patients with diabetes and on the other hand the complexity of physical activity behavior of this group is due to their problems, in this study, we intend to study the simultaneous use of intervention based on protection motivation theory along with the implementation *intention* on improvement of the physical activity level and physiological indicators of patients with type 2 diabetes.

## 2. Methods

### 2.1. Study Design and Participants

This experimental study was a field trial in 2018 on 126 patients with type 2 diabetes who were covered by Rafsanjan urban health centers. Potential participants were selected by random multistage cluster sampling. We randomly selected two centers from Rafsanjan's list of 8 health centers and randomly assigned one center to the intervention and one to the control group. We randomly selected patients with type 2 diabetes from these health centers, visited the health centers, and invited patient's participation in the study. The inclusion criteria were included age 18-65 years old, patients with a history of type 2 diabetes for at least one year, and being able to perform the recommended physical activity (based on The Physical Activity Readiness Questionnaire). Patients with complications such as diabetic retinopathy; diabetic foot; diseases causes disrupting in exercise, such as muscle, skeletal, and joint problems; systolic blood pressure (SBP) > 160 mm Hg and diastole blood pressure (DBP) > 100 mm Hg before exercise; and blood sugar > 300 Mg/dL or blood sugar < 70 Mg/dL were excluded from the study.

### 2.2. Sample Size and Sampling

The sample size was calculated by using the following formula and according to previous studies [17]. 59 patients were selected to be in each group.

(1) $$\begin{array}{c} n=\frac{2\sigma_{d}^{2}{\left(Z_{1-\left(\alpha /2\right)}+Z_{1-\beta }\right)}^{2}}{\delta^{2}}. \end{array}$$

**Figure 1:**
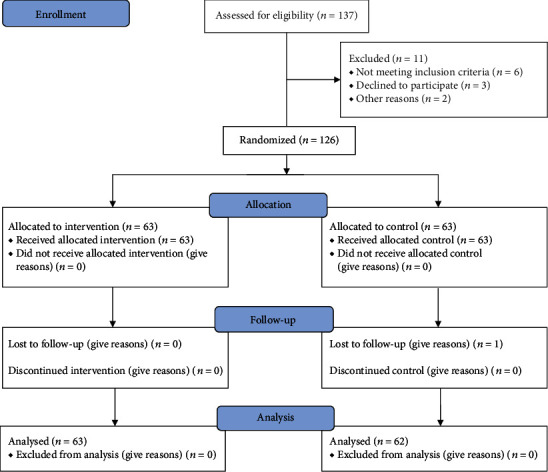
The flow diagram of the study.

Due to the possibility of losing the sample, 126 patients entered the study. 63 samples were considered in each group (Figure 1) to improve the study power.

### 2.3. Measurement

#### 2.3.1. Demographic Information

Demographic information of the participants included age, sex, education level, and income level.

#### 2.3.2. The Physical Activity Readiness Questionnaire (PAR-Q)

Readiness for performing physical activity was assessed by using the PAR-Q. The PAR-Q was completed by participants who planned to become “much more physically active.” This questionnaire has seven yes and no questions. If the answer is positive to one of the options, it is necessary to consult with a doctor in order to perform physical activity. The validity and reliability of the PAR-Q were examined previously (*r* = 0.99) [18]. In Iran, the study of Gholamnia-Shirvani et al. had an ICC = 0.85 [19].

#### 2.3.3. International Short Form Physical Activity Questionnaire (IPAQ)

In order to determine the level of physical activity, the IPAQ was used, which measures all physical activities in the workplace, sports activities, and daily activities of life. This standard questionnaire has been prepared by the World Health Organization, and its validity and reliability have been confirmed in different countries [20]. In Iran, the Persian version of this questionnaire has been used in many cases, and also, its validity has been confirmed [21]. The total score is calculated by summing the time and number of days of the week which have spent on moderate-intensity, high-intensity, and walking activities and converting them to METs (metabolic equivalent per minute).

#### 2.3.4. The Maximal Capacity for Oxygen Consumption by the Body during Maximal Exertion (VO2 max)

Rockport test is one of the simplest ways to assess heart and lung fitness in which a person's exercise capacity and maximum oxygen consumption are measured indirectly that can be performed for all age groups and even sedentary groups. In this test, a person is asked to walk a mile (1.6 km) as a brisk walk. After completing the path, the individuals were examined using a digital pulse oximetry device. Finally, the participant VO2 max will be calculated using the formula by taking into account the heart beat rate, time, age, and sex [22].

#### 2.3.5. Hemoglobin A1C

The amounts of HbA1c and blood lipids were determined using the same kit in the reference laboratory.

### 2.4. Intervention

The venue for the sessions was at the health centers, and the intervention group was divided into five groups of 10 and one group of 13 people based on the ability to attend classes in different hours. It should be noted that the interventions were done from 6 to 8 p.m. because of the proximity of the patients' sleeping hours and the lower rate of staff. In addition, a male researcher and a female one performed all interventions (for male and female participants).

The participants received protective motivational interventions for four sessions (Box 1) and implementation intention intervention for five sessions (Box 2) (for 45-60 minutes).

#### 2.4.1. Protective Motivational Interventions

Based on pretest scores and theoretical motivational structures, four intervention sessions were performed for the intervention group. In the first session, we tried to explain sensitivity and perceived intensity of the complications based on statistics and by the method of lecturing and showing films and diagrams which are caused by diabetes due to inactivity. The way of decreasing the effects of diabetes by exercising has been explained in the second session, as well as statistics and facts about this effect were provided based on the results of studies and the opinion of experts. The third session was about the basis of the training verbal persuasion to strengthen the sense of empowerment. The participants were also asked to share their experiences in order to improve their self-efficacy through exercise using an effective pattern of self-efficacy. The educational intervention in the fourth session was an attempt to reduce the costs and the perceived reward for exercise. Lectures, discussions with group members on perceived costs for exercise, and presentation of educational strategies were in order to minimize perceived costs, discussion, and exchange of views on perceived rewards, and presentation of valid statistics and facts and scientific recommendations were in order to reduce the perceived costs of the interventions of this training session (Box 1).

#### 2.4.2. Implementation Intention Intervention

At first, the necessity of implementing the intention and benefits was explained in this session. In the next step, they have been explained how to implement the intention. At this session, people were asked to state the obstacles that in their idea they are caused to prevent them from exercising (brainstorming). Individuals' views were listed on barriers to physical activity. People were asked to express and discuss strategies for overcoming obstacles. In the next step, people were asked to think about which of the barriers that they were talking about is the main barrier to exercising. Individuals were asked to list the obstacles which are caused to prevent them from exercising in the relevant form and find the way to solve those obstacles. In the next step, people were asked to list key situations that are appropriate to exercise and to write down these strategies in the form. After writing down these situations, they were asked to write that if they were in these situations, they would exercise or not. In the next step, people were asked to think about when they want to start the recommended exercise and write down the start time in the form (Box 2).

Participants in the control group received only routine care. Patients with type 2 diabetes should determine their level of effort for physical activity relative to their level of fitness write down in the respective table. The use and referral to their program must be reminded to patients once a month by phone and text message. Physical activity levels, VO2 max, HbA1c, and blood lipids were evaluated in two control and intervention groups before and three months after intervention.

### 2.5. Data Collection and Analysis

Sampling started after receiving the code of ethics as well as coordinating with the head of the health centers and the authorities of the care units and patients. The SPSS 18 software was used to analyze the data. *T*-test is generally used for normal distribution, and Mann–Whitney test is used for abnormal distribution. The pair *t*-test was also used for comparing the groups before and after the study, and Wilcoxon signed-rank test was used for abnormal distribution of data.

### 2.6. Ethical Considerations

This research has a code of ethics No. IR.RUMS.REC. 1395.72 with ID number of 95066 from Rafsanjan University of Medical Sciences. Before sampling, informed written consent was taken from diabetes patients, who were explained about the objectives of the study, confidentiality, and anonymity of the information and the voluntary participation in the study and voluntary withdrawal at any time.

## 3. Results

A total of 125 people participated in the study. One person was excluded from the study due to withdrawal from the plan (one sample from the control group). The results of the demographic study showed that most of the participants were women in the age range of 50-60 years. The two groups did not show a significant difference in terms of demographic characteristics (Table 1). The results of comparing the VO2 max in the control and intervention groups showed that the difference was not statistically significant, but the rate of changes in the intervention group was significantly higher. The results of the study on score in the two groups before and after the intervention showed that the VO2 max score after the intervention was significantly increased in the integration group compared to before the intervention (Table 2).

The results of the study on physical activity level (Mann–Whitney) showed that the intervention group had significantly more physical activity. The Wilcoxon test showed that the level of physical activity in the intervention group has been increased after the intervention (Table 3). The results of *t*-test showed that the amount of HbA1c was significantly lower in the intervention group. Comparison of the scores of each group before and after the intervention showed that the change in HbA1c in the integration group before and after the training intervention was significant, and it has been in the direction of reduction, while there was no significant difference in the control group (Table 4). Blood lipid tests showed that only the amount of cholesterol was significantly reduced before and after the intervention (*p* = 0.003). Other blood lipids did not show a significant difference before and after the intervention in the two groups (Table 5).

## 4. Discussion

The results of the present study showed that conducting educational intervention based on the combination of protection motivation theory and implementation intention was able to improve the level of physical activity in patients with type 2 diabetes. These results are consistent with similar studies in this field. The studies of Morowati et al. [23] and Plotnikoff et al. [24] have shown that structures of protection motivation theory were able to predict the behavior of physical activity. The protection motivation theory is one of the most important psychological theories that has been proven in order to help in accepting health-related behaviors such as physical activity [25]. According to the destructive consequences of sedentary lifestyle and taking advantage of this theory from the fear factor, it can play a role in promoting physical activity [26]. The effect of educational intervention based on the theory of protection motivation in promoting health protection behaviors has also been shown in studies of Khiyali et al. [27], Mirkarimi et al. [28], and Malmir et al. [29]. It seems that people with diabetes may not be aware of the risk of complications of inactivity in diabetes; thus, increasing the perceived threat can promote motivation and intention to perform physical activity. On the other hand, if people with diabetes believe that they are able to do physical activity and can reduce or delay the effects of diabetes by doing physical activity, this motivation will be even greater.

One hundred and fifty minutes of moderate-intensity aerobic exercise, with the heart rate reaching 60 to 80 percent of baseline, recommended for improving cardiopulmonary function and the VO2 max [30]. The combination of protection motivation theory and implementation intention theory was used in the present study, and many studies show that this intervention is more effective than intervention based on the theory of protection motivation alone. The results of studies by Zhang and Cooke [31], Mirkarimi et al. [28], and Dehdari et al. [32] show that the combination of these two theories in promoting physical activity behavior is more than the interventions only based on protection motivation. The implementation intention is effective in transforming intention into behavior in two ways. Avoid forgetting the subject is the first way. There is also a strong link between the planned situation and the intended behavior; therefore, the probability of starting the behavior increases [33]. The results of the present study showed that intervention could not cause a significant difference in the VO2 max in the two groups.

The recommended amount for improving cardiopulmonary function and the VO2 max 150 minutes of moderate-intensity aerobic activity so that the heart beat rate reaches 60-80% of the maximum heart beat rate [34]. In the present study, the majority of patients did not perform the recommended amount of physical activity and recommended intensity, and these actions may show the lack of difference between the two groups. In the intervention group, the amount of HbA1c was significantly reduced after the intervention. This is consistent with Najafipour's study, which showed that eight weeks of physical activity cause to reduce HbA1c levels [35]. However, the rate of change in HbA1c in our study was lower than (-0.34) in Najafipour's study (-0.06). Perhaps the reason is that the intensity of physical activity was higher in the Najafipour's study.

The results of the present study showed that there was no difference between blood lipid levels in the intervention group and the control group except for total cholesterol. However, Pedersen in his study on 13 meta-analyses studies has shown that blood lipid levels have been improved by exercise [36]. According to previous studies, HDL is the most important component of blood fats, which is likely to be enhanced by physical activity of 150 minutes per week with an intensity of 40-60% of maximum oxygen consumption. While other levels of lipids are also affected by increasing the intensity of physical activity [37], the average level of physical activity after the intervention shows that people did not have such a high level of physical activity to have a positive effect on blood lipids. Regarding the effect of intervention on blood cholesterol, it can be said that the performed interventions have caused to control the diet, and considering that cholesterol is more affected by diet, so this change does not seem to be due to the effects of promoting physical activity.

The present study is one of the limited studies that examined the effect of simultaneous intervention based on protection motivation theory and implementation intention to promote physical activity in patients with type 2 diabetes. Other strength of this study included that in addition to measuring the level of physical activity with the questionnaire, the physiological and hematological consequences of physical activity were also evaluated on improving the condition of diabetic patients such as VO2 max and blood lipids. However, this study also has some limitations, such as the three-month interval between the intervention and the subsequent test. Perhaps if this period will be longer, it is better to evaluate the effects of the intervention, especially on the physiological consequences. It is recommended to evaluate interventions based on the combination of this theory with the implementation of regular exercise programs and more monitoring to evaluate its effects on improving the physiological parameters of diabetic patients.

## 5. Conclusion

The results showed that the use of structured education based on the protection motivation theory and the implementation intention can be effective in encouraging patients to start physical activity and their success in performing the recommended physical activity. In the present study, we did not observe the effects of increasing the level of physical activity on indicators such as cardiovascular fitness as well as blood lipids. It is necessary to carry out more effective and intensive sports interventions in the next stages, with more supervision in this group of patients.

## Figures and Tables

**Box 1:**
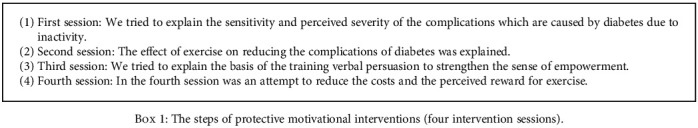
The steps of protective motivational interventions (four intervention sessions).

**Box 2:**
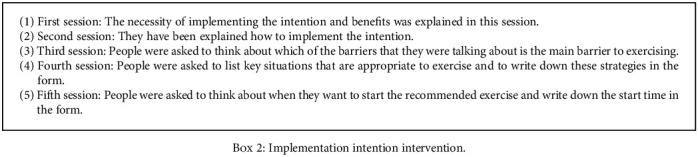
Implementation intention intervention.

**Table 1:** Demographic characteristics of participants of patients in both groups (*N* = 125).

Variable		Groups		*p*
		Intervention *N* (%)	Control *N* (%)	
Gender	Female	49 (77.8)	48 (78.7)	0.54
	Male	14 (22.2)	13 (21.3)	
Age	25-39	3 (4.8)	3 (4.8)	0.74
	40-49	14 (22.2)	9 (14.5)	
	50-59	41 (65.1)	44 (71.0)	
	60-65	5 (7.9)	6 (9.7)	
Education level	Primary	21 (33.3)	19 (30.6)	0.36
	Intermediate	27 (42.9)	28 (45.2)	
	Diploma and above	15 (23.8)	9 (14.5)	
Income level	Weak	17 (27)	25 (40.3)	0.24
	Moderate	44 (69.8)	36 (58.1)	
	Good	2 (3.2)	1 (1.6)	

**Table 2:** Comparison VO2 max in two control and intervention groups.

Variable	Stage	Intervention group	Control group	*p*
		Mean ± SD	Mean ± SD	
VO2 max	Before intervention	10.69 ± 20.42	8.69 ± 20.21	096
	After intervention	8.99 ± 22.87	8.88 ± 20.77	0.19
	*p*	0.0001	0.59	
Difference changes		1.44 ± 2.45	1.08 ± 0.56	0.0001

**Table 3:** Physical activity level score based on MET in two control and intervention groups.

Variable	Stage	Intervention group	Control group	*p*
		Median (IQR^∗^)	Median (IQR^∗^)	
MET level of physical activity	Before intervention	396 (450)	297 (396)	0.97
	After intervention	495 (297)	396 (396)	0.02
	*p*	0.001	0.89	
Difference changes		198 (180)	198 (0)	0.001

IQR: interquartile range.

**Table 4:** Comparison HbA1c in both intervention and control groups.

Variable	Stage	Intervention group	Control group	*p*
		Mean ± SD	Mean ± SD	
HbA1c	Before intervention	0.87 ± 7.07	0.62 ± 7.37	0.57
	After intervention	0.80 ± 6.86	0.52 ± 7.40	0.0001
	*p*	0.0001	0.67	
Difference changes		0.33 ± −0.21	0.38 ± 0.06	0.046

**Table 5:** Comparison blood lipid levels in intervention and control groups.

Variable	Stage	Intervention group	Control group	*p*
		Mean ± SD	Mean ± SD	
HDL levels	Preintervention	8.7 ± 44.29	8.32 ± 42.95	0.38
	Postintervention	0.80 ± 44.83	8.47 ± 44.83	0.15
	*p*	0.11	0.83	
LDL levels	Preintervention	25.22 ± 125.11	23.82 ± 112.01	0.004
	Postintervention	27.4 ± 121.61	23.15 ± 115.06	0.17
	*p*	0.15	0.21	
Cholesterol levels	Preintervention	38.31 ± 173.06	31.34 ± 174.52	0.57
	Postintervention	40.90 ± 164.77	25.65 ± 173.38	0.10
	*p*	0.003	0.50	
Triglyceride levels	Preintervention	56.31 ± 156.58	51.97 ± 176.83	0.038
	Postintervention	53.44 ± 153.98	25.65 ± 174.38	0.018
	*p*	0.32	0.38	

## Data Availability

Data are available by contacting with the corresponding author by email.

## References

[1] Saeedi P., Petersohn I., Salpea P. (2019). Global and regional diabetes prevalence estimates for 2019 and projections for 2030 and 2045: Results from the International Diabetes Federation Diabetes Atlas, 9th edition. *Diabetes Research and Clinical Practice*.

[2] Esteghamati A., Etemad K., Koohpayehzadeh J. (2014). Trends in the prevalence of diabetes and impaired fasting glucose in association with obesity in Iran: 2005–2011. *Diabetes Research and Clinical Practice*.

[3] Colberg S. R., Sigal R. J., Yardley J. E. (2016). Physical activity/exercise and diabetes: a position statement of the American Diabetes Association. *Diabetes Care*.

[4] Hamasaki H. (2016). Daily physical activity and type 2 diabetes: a review. *World Journal of Diabetes*.

[5] Yang D., Yang Y., Li Y., Han R. (2019). Physical exercise as therapy for type 2 diabetes mellitus: from mechanism to orientation. *Annals of Nutrition and Metabolism*.

[6] Silva D. A. S., Naghavi M., Duncan B. B., Schmidt M. I., de Fatima Marinho de Souza M., Malta D. C. (2019). Physical inactivity as risk factor for mortality by diabetes mellitus in Brazil in 1990, 2006, and 2016. *Diabetology & Metabolic Syndrome*.

[7] American Diabetes Association (2016). 3. Foundations of care and comprehensive medical evaluation. *Diabetes Care*.

[8] Sanghamitra P., Eunice L., Sandipana P., Shayma D., Pranab M. (2019). Type 2 diabetes and physical activity: barriers and enablers to diabetes control in Eastern India. *Primary Health Care Research & Development*.

[9] Al-Zalabani A. H., Al-Hamdan N. A., Saeed A. A. (2015). The prevalence of physical activity and its socioeconomic correlates in Kingdom of Saudi Arabia: a cross-sectional population-based national survey. *Journal of Taibah University Medical Sciences*.

[10] Pirsaheb M., Almasi A., Rezaee M. (2010). The special health education course effects on knowledge, attitude and practice of preparation, distribution and sale centers food staff in Kermanshah. *Iranian Journal of Health and Environment*.

[11] Kok G. (2014). A practical guide to effective behavior change: how to apply theory- and evidence-based behavior change methods in an intervention. *The European Health Psychologist*.

[12] Naito M., O'Callaghan F. V., Morrissey S. (2009). Understanding women’s mammography intentions: a theory-based investigation. *Women & Health*.

[13] Plotnikoff R. C., Trinh L. (2010). Protection motivation theory: is this a worthwhile theory for physical activity promotion?. *Exercise and Sport Sciences Reviews*.

[14] da Silva M. A. V., São-João T. M., Brizon V. C., Franco D. H., Mialhe F. L. (2018). Impact of implementation intentions on physical activity practice in adults: a systematic review and meta-analysis of randomized clinical trials. *PLoS One*.

[15] Gollwitzer P. M. (1999). Implementation intentions: strong effects of simple plans. *American Psychologist*.

[16] Scholz U., Sniehotta F. F., Burkert S., Schwarzer R. (2007). Increasing physical exercise levels: age-specific benefits of planning. *Journal of Aging and Health*.

[17] Prestwich A., Perugini M., Hurling R. (2010). Can implementation intentions and text messages promote brisk walking? A randomized trial. *Health Psychology*.

[18] Warburton D. E., Bredin S. S., Jamnik V. K., Gledhill N. (2011). Validation of the PAR-Q+ and ePARmed-X+. *The Health & Fitness Journal of Canada*.

[19] Gholamnia-Shirvani Z., Ghofranipour F., Gharakhanlou R., Kazemnejad A. (2018). “Women and active life”: an extended TPB-based multimedia software to boost and sustain physical activity and fitness of Iranian women. *Women & Health*.

[20] Wendel-Vos G. W., Schuit A. J., Saris W. H., Kromhout D. (2003). Reproducibility and relative validity of the short questionnaire to assess health-enhancing physical activity. *Journal of Clinical Epidemiology*.

[21] Emami R. S., Ardebili H. E., Golestan B. (2010). Effect of a health education intervention on physical activity knowledge, attitude and behavior in health volunteers. *Hayat*.

[22] Hoffman J. (2006). *Norms for fitness, performance, and health*.

[23] Morowatisharifabad M. A., Abdolkarimi M., Asadpour M., Fathollahi M. S., Balaee P. (2018). The predictive effects of protection motivation theory on intention and behaviour of physical activity in patients with type 2 diabetes. *Open access Macedonian journal of medical sciences*.

[24] Plotnikoff R. C., Rhodes R. E., Trinh L. (2009). Protection motivation theory and physical activity: a longitudinal test among a representative population sample of Canadian adults. *Journal of Health Psychology*.

[25] Plotnikoff R. C., Lippke S., Trinh L., Courneya K. S., Birkett N., Sigal R. J. (2010). Protection motivation theory and the prediction of physical activity among adults with type 1 or type 2 diabetes in a large population sample. *British Journal of Health Psychology*.

[26] Wong T. S., Gaston A., DeJesus S., Prapavessis H. (2016). The utility of a protection motivation theory framework for understanding sedentary behavior. *Health Psychology and Behavioral Medicine*.

[27] Khiyali Z., Ghahremani L., Kaveh M. H., Keshavarzi S. (2017). The effect of an educational program based on protection motivation theory on pap smear screening behavior among women referring to health centers in Fasa. *Journal of Education and Community Health*.

[28] Mirkarimi K., Mostafavi F., Eshghinia S., Vakili M. A., Ozouni-Davaji R. B., Aryaie M. (2015). Effect of motivational interviewing on a weight loss program based on the protection motivation theory. *Iranian Red Crescent Medical Journal*.

[29] Malmir S., Barati M., Khani Jeihooni A., Bashirian S., Hazavehei S. M. M. (2018). Effect of an educational intervention based on protection motivation theory on preventing cervical cancer among marginalized women in west Iran. *Asian Pacific Journal of Cancer Prevention: APJCP*.

[30] Conner M., Norman P. (2015). Predicting and changing health behaviour: research and practice with social cognition models. *Maidenhead*.

[31] Zhang Y., Cooke R. (2012). Using a combined motivational and volitional intervention to promote exercise and healthy dietary behaviour among undergraduates. *Diabetes Research and Clinical Practice*.

[32] Dehdari T., Hassani L., Hajizadeh E., Shojaeizadeh D., Nedjat S., Abedini M. (2014). Effects of an educational intervention based on the protection motivation theory and implementation intentions on first and second pap test practice in Iran. *Asian Pacific Journal of Cancer Prevention*.

[33] Webb T. L., Sheeran P. (2007). How do implementation intentions promote goal attainment? A test of component processes. *Journal of Experimental Social Psychology*.

[34] Sigal R. J., Kenny G. P., Wasserman D. H., Castaneda-Sceppa C. (2004). Physical activity/exercise and type 2 diabetes. *Diabetes Care*.

[35] Najafipour F., Mobasseri M., Yavari A. (2017). Effect of regular exercise training on changes in HbA1c, BMI and VO2max among patients with type 2 diabetes mellitus: an 8-year trial. *BMJ Open Diabetes Research and Care*.

[36] Pedersen B. K., Saltin B. (2006). Evidence for prescribing exercise as therapy in chronic disease. *Scandinavian Journal of Medicine & Science in Sports*.

[37] Nybo L., Sundstrup E., Jakobsen M. D. (2010). High-intensity training versus traditional exercise interventions for promoting health. *Medicine & Science in Sports & Exercise*.

